# Supplementary material for: Transcultural adaptation and validation of the questionnaire “Urgency, Weak stream, Incomplete emptying and Nocturia (UWIN)” for the Brazilian Portuguese
Source: PeerJ. 2020 May 25;8:e9039. doi: 10.7717/peerj.9039 (PMC7255330; doi:10.7717/peerj.9039)
Supplement: Supplemental Information 2 [file peerj-08-9039-s002.docx]

- Flowmax = Maxime Flow

- IPSS 1 = Question 1 IPSS
- IPSS 2 = Question 2 IPSS
- .......
- IPSS 7 = Question 7

- SCOIPSS = Total score IPSS

- UWIN 1 = Question 1
- ......
- UWIN4 = Question 4

- QOL = quality of life

- tap= time of aplication in minutes

- ClassIPSS
  - 0=Mild
  - 1=Moderate
  - 2=Severe

- Education = years of education

- mod = APLICATION MODE
  - 0 = Questionnaire
  - 1 = Interview
