# Supplementary material for: Transcultural adaptation and validation of the questionnaire “Urgency, Weak stream, Incomplete emptying and Nocturia (UWIN)” for the Brazilian Portuguese
Source: PeerJ. 2020 May 25;8:e9039. doi: 10.7717/peerj.9039 (PMC7255330; doi:10.7717/peerj.9039)
Supplement: Supplemental Information 3 [file peerj-08-9039-s003.docx]

|  | **Nunca** | **Menos da metade das vezes** | **Aproximadamente metade das vezes** | **Mais da metade das vezes** |
| --- | --- | --- | --- | --- |
| **No último mês, quantas vezes foi difícil segurar a vontade de urinar?** | **( )** | **( )** | **( )** | **( )** |
| **No último mês, quantas vezes você achou que o jato de urina estava fraco?** | **( )** | **( )** | **( )** | **( )** |
| **No último mês, quantas vezes você teve a sensação de não ter esvaziado completamente a bexiga após terminar de urinar?** | **( )** | **( )** | **( )** | **( )** |
| **No último mês, quantas vezes em média você teve que levantar-se a noite para urinar?** | **0 vezes**  **( )** | **1 a 2 vezes**  **( )** | **3 vezes**  **( )** | **4 vezes ou mais**  **( )** |

|  | **Satisfeito** | **Nem satisfeito/ Nem insatisfeito** | **Insatisfeito** |
| --- | --- | --- | --- |
| **Caso você tivesse que viver a vida com os sintomas urinários que você tem atualmente, como você se sentiria?** | **( )** | **( )** | **( )** |
