# Supplementary material for: Transcultural adaptation and validation of the questionnaire “Urgency, Weak stream, Incomplete emptying and Nocturia (UWIN)” for the Brazilian Portuguese
Source: PeerJ. 2020 May 25;8:e9039. doi: 10.7717/peerj.9039 (PMC7255330; doi:10.7717/peerj.9039)
Supplement: Supplemental Information 4 [file peerj-08-9039-s004.docx]

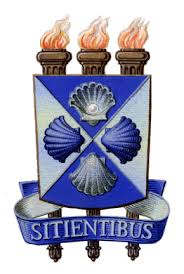

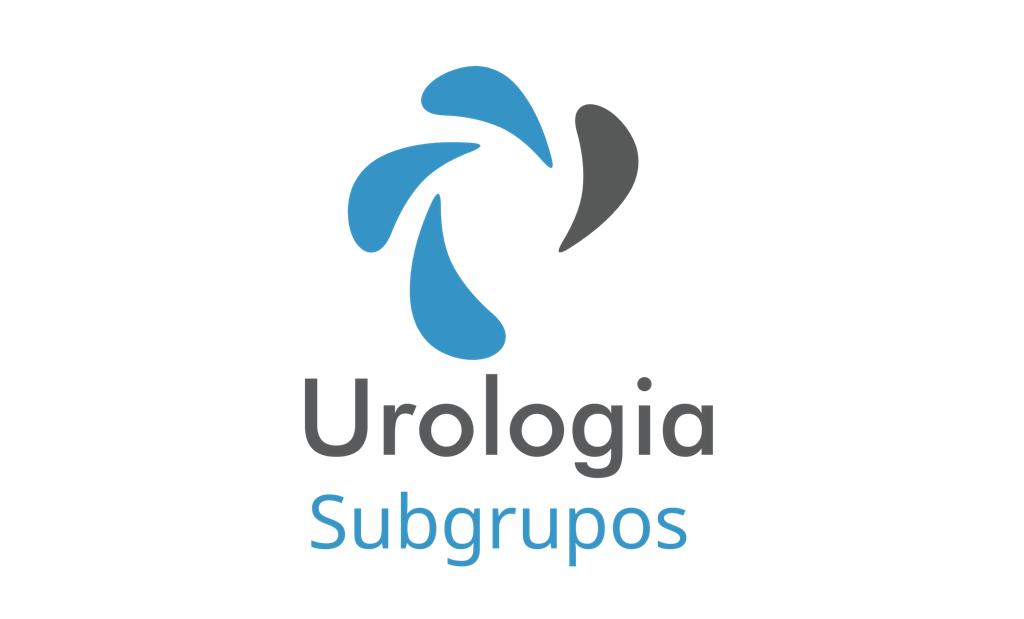


**UWIN – Br**

**GUIA DO USUÁRIO**

**“Esse questionário tem por objetivo identificar possíveis sintomas urinários apresentados por você. ”**

**1 – Para responder as perguntas, pense na ocorrência dos sintomas no último mês;**

**2 - Leia atentamente cada pergunta e assinale apenas uma alternativa - a que melhor representa a frequência com que o sintoma foi percebido por você.**


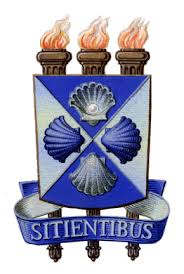

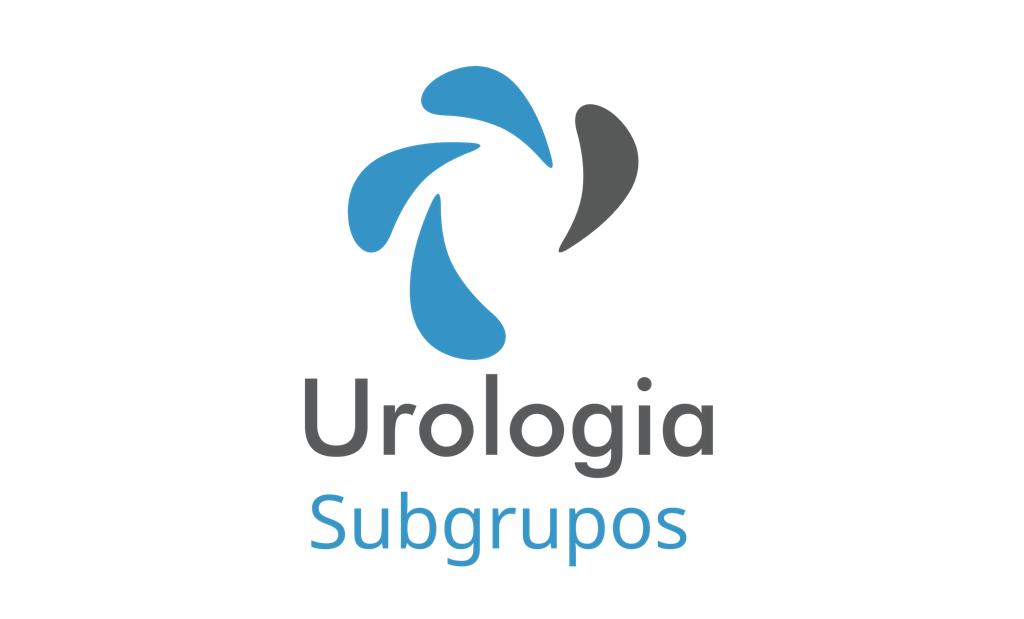


**UWIN – Br**

**GUIA DO APLICADOR**

**“Esse questionário tem por objetivo identificar possíveis sintomas urinários apresentados por homens. ”**

**1 – O questionário deverá ser preferencialmente auto aplicado (o próprio participante responde);**

**2 – Interpretação:**

- **O questionário é composto por 4 perguntas referentes aos sintomas com quatro opções de respostas (cada opção de resposta receberá pontuação de 0 a 3) e uma questão referente a qualidade de vida com três opções de respostas;**
- **O Score é classificado de acordo com a somatória da pontuação das respostas do participante para as perguntas referentes aos sintomas;**
- **Classificação: Score 0 a 3 leve;**
- **4 a 7 moderado;**
- **8 a 12 grave.**
